# Supplementary material for: A comprehensive study of cognitive control in healthy aging
Source: Commun Med (Lond). 2025 Nov 24;6:2. doi: 10.1038/s43856-025-01239-1 (PMC12764825; doi:10.1038/s43856-025-01239-1)
Supplement: Supplementary file 2 — Supplementary information [file 43856_2025_1239_MOESM2_ESM.pdf]

A Comprehensive Study on Cognitive Control in Healthy Aging

Supplementary Information

Sarah De Pue*, Hans Stuyck, Céline R. Gillebert, Eva Dierckx & Eva Van den Bussche

*Corresponding author

E-mail: sarah.depue@kuleuven.be

Supplementary Table 1

Overview of participants' demographic and general characteristics measured per Age Group for the older adults.

			60-69	70-79	80+
			N	*N*	*N*
Highest educational level	No secondary school diploma		9	11	10
	Secondary school diploma		14	26	19
	University or high school degree		52	28	28
	Other		5	17	12
Work situation	Retired		63	80	68
	Employed		12	1	1
	Other (e.g., disabled, looking for a job, early retirement or retirement with part-time job)		6	1	0
Monthly family net income (in €)	501-1000		1	0	0
	1001-1500		5	7	12
	1501-2000		11	11	15
	2001-2500		8	23	16
	2501-3000		9	8	3
	3001-3500		10	12	9
	3501-4000		9	4	5
	Above 4000		22	10	1
	I can't or do not want to answer this question		5	7	8
Computer use	Have you already used a computer?	No	1	7	12
		Yes	79	75	57
	How often have you already used a computer?	Very rarely	0	2	0
		Rarely	3	2	2
		Sometimes	6	10	10
		Often	20	29	27
		Very often	50	32	18
	How much experience do you have with a computer?	Very little	1	1	1
		Little	8	9	14
		Average	30	40	34
		Much	24	18	5
		Very much	16	7	3
	How would you rate your own computer skills	Beginner	9	11	14
		Average	51	51	38
		Advanced	19	13	5
Subjective cognitive complaints	Yes		7	9	9
	No		73	73	60

Supplementary Table 2

Overview of diseases that could affect cognitive functioning indicated by participants.

	Young adults	60-69	70-79	80+
	N	*N*	*N*	*N*
None	72	67	73	61
Learning disorder: dyslexia	1	0	0	0
ADD	1	0	0	0
DPDR	1	0	0	0
Burnout	0	2	2	0
Sleep apnea	0	1	0	0
Jaundice	0	0	0	2
Cancer (more than 2 years ago)	0	4	1	1
TIA/CVA/stroke	0	1	0	3
Depression	0	0	1 (chronical)	1 (50 years ago)
Concussion/whiplash	0	4	0	0
Epilepsy	0	1	0	1
Heart disease	0	1	2	0
Ménière’s disease	0	0	1	0
Diabetes	0	0	2	0

Supplementary Table 3

Comparison of models with different random structures per task.

	Random factor structure	npar	AIC	Log lik.	X^2	*p*
Flanker 47C RT	1. Participant (intercept)	10	-243078	121591		
	**2. Participant (intercept), Congruency (slope)**	12	-243209	121616	50.03	<.001
Flanker 47C ERR	**1. Participant (intercept)**	9	8377.9	-4179.9		
	2. Participant (intercept), Congruency (slope)	11	8379.3	-4178.7	2.55	0.28
Plus-Minus RT	1. Participant (intercept)	10	9418.1	-4699		
	**2. Participant (intercept), Switch (slope)**	12	9349.2	-4662.6	72.85	<.001
Plus-Minus ERR	1. Participant (intercept)	9	2610	-1296		
	**2. Participant (intercept), Switch (slope)**	11	2452.2	-1201.6	188.79	<.001
AX-CPT RT	1. Participant (intercept)	18	693137	-346550		
	**2. Participant (intercept), Trial Type (slope)**	27	690235	-345090	2920.20	<.001
AX-CPT ERR	1. Participant (intercept)	17	19801	-9883.4		
	**2. Participant (intercept), Trial Type (slope)**	26	19569	-9758.3	250.32	<.001
Flanker PC RT	1. Participant (intercept)	26	-687736	343894		
	2. Participant (intercept), Congruency (slope)	28	-688141	344098	408.58	<.001
	3. Participant (intercept), Block (slope)	31	-691882	345972	3746.83	<.001
	**4. Participant (intercept), Congruency (slope), Block (slope)**	35	-692040	346055	166.90	<.001
	5. Participant (intercept), Block x Congruency (slope)			Failed to converge		
Flanker PC ERR	1. Participant (intercept)	25	22875	-11412		
	**2. Participant (intercept), Congruency (slope)**	27	22854	-11400	25.02	<.001
	3. Participant (intercept), Block (slope)	30	22874	-11407	0.00	1.00
	4. Participant (intercept), Congruency (slope), Block (slope)			Failed to converge		
	5. Participant (intercept), Block x Congruency (slope)			Failed to converge		

Note: For the Go/No-Go and N-Back, only one random structure was possible (i.e. random intercept Participant), so no model comparisons were necessary. Bold text indicates the best fitting model.

Supplementary Methods

Below you can find an example equation of the LMM for the reaction times on the 47C block of the Flanker, used to study inhibition. This equation can be extended to other models in the manuscript as well.

Level 1: $Y_{ij} = \beta_{0j} + \beta_1 Contrast1_{ij} + \beta_2 Contrast2_{ij} + \beta_3 Contrast3_{ij} + \beta_{4j} Conguency_{ij} + \beta_5 Contrast1 * Congruency_{ij} + \beta_6 Contrast2 * Congruency_{ij} + \beta_7 Contrast3 * Congruency_{ij} + \varepsilon_{ij}$

Level 2: $\beta_{0j} = \beta_0 + u_{0j}$

$\beta_{4j} = \beta_4 + u_{4j}$

In this linear mixed effects model, Y_{ij} represents the reaction time for participant *j* on the *i*-th trial, modeling trial-level variations in reaction times. The level 1 model includes fixed effects for the three age group contrasts (i.e., Contrast 1: young adults vs. 80+; Contrast 2: 60-69 vs. 80+; and Contrast 3: 70-79 vs. 80+), congruency (i.e., congruent vs. incongruent), and their interaction terms. The term ε_{ij} represents the residual error for trial *i* of participant *j*, capturing trial-level variability that could not be explained by the fixed and random effects. The intercept and the slope for congruency are allowed to vary across participants, as specified in level 2. Specifically, each participant has a unique intercept β_{0j} and a unique slope for congruency β_{4j}, modeled as deviations (i.e., u_{0j} and u_{4j}, respectively) from the overall intercept β_0 and slope for congruency β_4.

Supplementary Table 4

Pairwise comparisons with Tukey correction for multiple comparisons for the interaction between Trial Type and Age Group for reaction times on the AX-CPT.

Age Group	Contrast	*t*	*df*	*p*	Cohen's *d*
Young adults	AY (576ms) vs. AX (432ms)	12.97	272	*p*<.001	-0.98
	BX (403ms) vs. AX (432ms)	-2.17	277	*p*=.13	-
	BY (383ms) vs. AX (432ms)	-7.80	279	*p*<.001	0.34
	**BX (403ms) vs. AY (576ms)**	**-11.43**	**274**	***p*<.001**	**1.18**
	BY (383ms) vs. AY (576ms)	-14.76	278	*p*<.001	1.32
	BY (383ms) vs. BX (403ms)	-2.45	255	*p*=.071	-
60-69	AY (719ms) vs. AX (557ms)	15.27	269	*p*<.001	-1.10
	BX (563ms) vs. AX (557ms)	0.44	277	*p*=.97	-
	BY (547ms) vs. AX (557ms)	-1.09	283	*p*=.70	-
	**BX (563ms) vs. AY (719ms)**	**-10.78**	**272**	***p*<.001**	**1.06**
	BY (547ms) vs. AY (719ms)	-13.78	278	*p*<.001	1.18
	BY (547ms) vs. BX (563ms)	-2.12	256	*p*=.15	-
70-79	AY (777ms) vs. AX (574ms)	18.93	273	*p*<.001	-1.38
	BX (588ms) vs. AX (574ms)	1.08	277	*p*=.70	-
	BY (554ms) vs. AX (574ms)	-2.03	284	*p*=.14	-
	**BX (588ms) vs. AY (777ms)**	**-12.91**	**274**	***p*<.001**	**1.29**
	BY (554ms) vs. AY (777ms)	-17.63	281	*p*<.001	1.52
	BY (554ms) vs. BX (588ms)	-4.39	257	*p*<.001	0.23
80+	AY (843ms) vs. AX (633ms)	17.59	276	*p*<.001	-1.43
	BX (622ms) vs. AX (633ms)	-0.75	281	*p*=.88	-
	BY (601ms) vs. AX (633ms)	-2.92	287	*p*=.020	0.22
	**BX (622ms) vs. AY (843ms)**	**-13.55**	**279**	***p*<.001**	**1.51**
	BY (601ms) vs. AY (843ms)	-17.17	286	*p*<.001	1.65
	BY (601ms) vs. BX (622ms)	-2.41	268	*p*=.078	-

Note. Effect sizes (Cohen's *d*) are only reported for significant comparisons (*p*<.05). In bold are the critical comparisons regarding proactive vs. reactive control: performance on AY vs. BX trials.

Supplementary Table 5

Pairwise comparisons with Tukey correction for multiple comparisons for the interaction between Age Group and Trial Type for reaction times on the AX-CPT.

Trial Type	Contrast	*t*	*df*	*p*	Cohen's *d*
AX	Group 2 (557ms) vs. Group 1 (432ms)	7.55	288	*p*<.001	-0.85
	Group 3 (574ms) vs. Group 1 (432ms)	8.53	288	*p*<.001	-0.97
	Group 4 (633ms) vs. Group 1 (432ms)	11.43	288	*p*<.001	-1.37
	Group 3 (574ms) vs. Group 2 (557ms)	1.03	288	*p*=.73	-
	Group 4 (633ms) vs. Group 2 (557ms)	4.39	288	*p*<.001	-0.51
	Group 4 (633ms) vs. Group 3 (574ms)	3.41	288	*p*=.0042	-0.40
**AY**	**Group 2 (719ms) vs. Group 1 (576ms)**	**6.08**	**284**	***p*<.001**	**-0.97**
	**Group 3 (777ms) vs. Group 1 (576ms)**	**8.50**	**285**	***p*<.001**	**-1.37**
	**Group 4 (843ms) vs. Group 1 (576ms)**	**10.72**	**286**	***p*<.001**	**-1.82**
	**Group 3 (777ms) vs. Group 2 (719ms)**	**2.51**	**284**	***p*=.061**	**-**
	**Group 4 (843ms) vs. Group 2 (719ms)**	**5.09**	**285**	***p*<.001**	**-0.85**
	**Group 4 (843ms) vs. Group 3 (777ms)**	**2.71**	**286**	***p*=.035**	**-0.45**
**BX**	**Group 2 (563ms) vs. Group 1 (403ms)**	**5.89**	**283**	***p*<.001**	**-1.09**
	**Group 3 (588ms) vs. Group 1 (403ms)**	**6.79**	**283**	***p*<.001**	**-1.26**
	**Group 4 (622ms) vs. Group 1 (403ms)**	**7.59**	**285**	***p*<.001**	**-1.49**
	**Group 3 (588ms) vs. Group 2 (563ms)**	**0.94**	**283**	***p*=.78**	**-**
	**Group 4 (622ms) vs. Group 2 (563ms)**	**2.08**	**285**	***p*=.16**	**-**
	**Group 4 (622ms) vs. Group 3 (588ms)**	**1.19**	**285**	***p*=.63**	**-**
BY	Group 2 (547ms) vs. Group 1 (383ms)	7.28	284	*p*<.001	-1.12
	Group 3 (554ms) vs. Group 1 (383ms)	7.59	284	*p*<.001	-1.17
	Group 4 (601ms) vs. Group 1 (383ms)	9.14	285	*p*<.001	-1.48
	Group 3 (554ms) vs. Group 2 (547ms)	0.34	285	*p*=.99	-
	Group 4 (601ms) vs. Group 2 (547ms)	2.32	286	*p*=.096	-
	Group 4 (601ms) vs. Group 3 (554ms)	1.99	286	*p*=.19	-

Note. Effect sizes (Cohen's *d*) are only reported for significant comparisons (*p*<.05). In bold are the critical comparisons regarding proactive vs. reactive control: performance on AY and BX trials.

Supplementary Table 6

Pairwise comparisons with Tukey correction for multiple comparisons for the interaction between Trial Type and Age Group for error rates on the AX-CPT.

Age Group	Contrast	*Z*	*p*	Cohen's *d*
Young adults	AY (10.03%) vs. AX (1.76%)	12.09	*p*<.001	-1.83
	BX (4.45%) vs. AX (1.76%)	5.38	*p*<.001	-0.96
	BY (1.78%) vs. AX (1.76%)	0.078	*p*=1.00	-
	**BX (4.45%) vs. AY (10.03%)**	**-4.39**	***p*<.001**	**0.87**
	BY (1.78%) vs. AY (10.03%)	-8.34	*p*<.001	1.82
	BY (1.78%) vs. BX (4.45%)	-4.60	*p*<.001	0.94
60-69	AY (5.06%) vs. AX (0.97%)	9.79	*p*<.001	-1.69
	BX (2.07%) vs. AX (0.97%)	3.66	*p*=.0014	-0.77
	BY (1.92%) vs. AX (0.97%)	3.58	*p*=.0020	-0.69
	**BX (2.07%) vs. AY (5.06%)**	**-3.99**	***p*<.001**	**0.92**
	BY (1.92%) vs. AY (5.06%)	-4.53	*p*<.001	1.00
	BY (1.92%) vs. BX (2.07%)	-0.36	*p*=.98	-
70-79	AY (7.09%) vs. AX (1.81%)	9.08	*p*<.001	-1.42
	BX (2.68%) vs. AX (1.81%)	2.08	*p*=.16	-
	BY (2.22%) vs. AX (1.81%)	1.14	*p*=.67	-
	**BX (2.68%) vs. AY (7.09%)**	**-4.71**	***p*<.001**	**1.02**
	BY (2.22%) vs. AY (7.09%)	-5.71	*p*<.001	1.21
	BY (2.22%) vs. BX (2.68%)	-0.93	*p*=.79	-
80+	AY (8.21%) vs. AX (1.68%)	9.59	*p*<.001	-1.65
	BX (3.74%) vs. AX (1.68%)	3.97	*p*<.001	-0.82
	BY (3.26%) vs. AX (1.68%)	3.70	*p*=.0013	-0.68
	**BX (3.74%) vs. AY (8.21%)**	**-3.62**	***p*=.0017**	**0.83**
	BY (3.26%) vs. AY (8.21%)	-4.52	*p*<.001	0.98
	BY (3.26%) vs. BX (3.74%)	-0.70	*p*=.90	-

Note. Effect sizes (Cohen's *d*) are only reported for significant comparisons (*p*<.05). In bold are the critical comparisons regarding proactive vs. reactive control: performance on AY vs. BX trials.

Supplementary results

Age differences in proactive and reactive control

AX-CPT. Post-hoc comparisons for the main effect of Trial Type for RTs showed that participants were significantly slower on the AY trials (*M*=729ms), followed by AX (*M*=549ms), BX (*M*=544ms) and BY trials (*M*=521ms). All comparisons were significant ($p<.001$), except for the difference between AX and BX trials ($p=.87$).

Post-hoc comparisons for the main effect of Trial Type for ERR showed that participants made significantly more errors on the AY trials (*M*=7.39%;) compared to the AX (*M*=1.51%; *OR*=5.20; $Z=18.05$, $p<.001$, $d=-1.65$), BX (*M*=3.11%; *OR*=0.40; $Z=-7.13$, $p<.001$, $d=0.91$) and BY trials (*M*=2.23%; *OR*=0.29; $Z=-9.50$, $p<.001$, $d=1.25$). Moreover, performance was better on AX trials compared to BX (*OR*=2.09; $Z=6.23$, $p<.001$, d=-0.74) and BY trials (*OR*=1.49; $Z=3.31$, $p=.0052$, d=-0.40). There was no significant difference between BX and BY trials ($p=.070$).

Flanker with proportion congruency manipulation.

Significant two-way interactions were only discussed here in the supplementary materials, as the interpretation of these effects was difficult due to the presence of the significant three-way interaction. Significant interaction effects between Age Group and Congruency and Block and Congruency were further explored by calculating the congruency effect (incongruent-congruent) based on the predicted means and comparing this difference score with a one-way ANOVA between groups and blocks with Tukey’s corrections for multiple comparisons.

Post-hoc comparisons for the main effect of Block for RTs showed that participants were significantly slower on the 87C block (*M*=646ms), compared to the 47C (*M*=635ms, $t(317)=3.31$, $p=.0030$, $d=-0.087$) and 67C blocks (*M*=632ms, $t(322)=3.98$, $p<.001$, $d=-0.11$). The other contrast was not significant ($p=.57$). Regarding the interaction effect between Congruency and Age Group, based on the descriptives, CE appeared larger for young adults (*M*=39ms) and the oldest age cohort (*M*=37ms) compared to the 60-69 and 70-79 age cohorts (*M*=32ms for both groups). However, post-hoc comparisons were not significant ($p\geq.31$). For the interaction between Block and Congruency, the

CE was calculated and compared between blocks. Results showed that the CE became significantly smaller with increasing amount of incongruent trials ($M_{87\%C}$ =69.60ms, $M_{67\%C}$=38.43ms, $M_{47\%C}$ =20.00ms, all p<.001), i.e. the proportion congruency effect (PCE).

Regarding the interaction effect between Block and Age Group for ERR, pairwise comparisons showed a significant difference in errors between blocks only for the older adults aged 60-69 and 80+. For the 60-69 group, significantly more errors were made on the 67C block (*M*=1.36%) compared to the 47C block (*M*=0.93%, *OR*=1.47, *Z*=3.22, *p*=.0037, *d*=-0.39). For the 80+ group, significantly more errors were made on the 87C block (*M*=2.04%) compared to the 67C (*M*=1.21%, *OR*=1.70, *Z*=3.97, *p*<.001, *d*=-0.53) and 47C block (*M*=1.48%, *OR*=1.38, *Z*=2.58, *p*=.027, *d*=-0.33). Moreover, whereas for all blocks young adults always performed worse than older adults (all *p*<.001), older adults aged 80+ performed significantly worse than older adults aged 60-69 only for the 47C (M_{60-69} =0.89%, M_{80+}=1.59%, *OR*=1.60, *Z*=2.62, *p*=.044, *d*=-0.47) and 87C (M_{60-69} =1.12%, M_{80+}=1.98%, *OR*=1.78, *Z*=2.91, *p*=.019, *d*=-0.58) blocks. Studying the interaction effect between Congruency and Age Group, young adults (*M*=1.58%) had a significantly larger CE than older adults (M_{60-69} =0.47%, M_{70-79} =0.33%, M_{80+}=0.43%), indicating a worse inhibition (all *p*≤.001). The differences in CE between the older adults were not significant (*p*≥.91). For the interaction between Block and Congruency, CEs were compared between blocks. With increasing proportion of incongruent trials, the CE became significantly smaller ($M_{87\%C}$ =1.44%, $M_{67\%C}$=0.77%, $M_{47\%C}$ =0.46%, *p*≤.001), i.e. the PCE.

Finally, for the three-way interaction between Block and Congruency and Age Group, the main manuscript already reports and compares the PCE between age groups. Here we further explore this interaction by also comparing the CE between age groups for each block separately, using one-way ANOVA with Tukey correction for multiple comparisons. Regarding RTs, for the 87C block, the CE did not differ significantly between age groups (*p*=.051). For the 67C block, the CE was significantly larger in the 80+ group (*M*=49ms) compared to the other age groups (M_{YA}=37ms, M_{60-69}=36ms, M_{70-79}=35ms; all *p*<.001). Finally, for the 47C block, the CE was larger in young adults

(*M*=23ms) compared to the 60-69 (*M*=12ms, *p*<.001) and 70-79 group (*M*=17ms, *p*=.030). Moreover, the CE was significantly larger in the 80+ group (*M*=21ms) compared to the 60-69 group (*M*=12ms, *p*<.001). Regarding error rates, for the 87C block, the CE was significantly larger in young adults (*M*=2.88%) compared to the older age groups ($M_{60\text{-}69}$=0.27%, $M_{70\text{-}79}$=1.32%, M_{80+}=1.40%; all *p*<.001). Moreover, the CE was significantly smaller in the 60-69 group compared to the oldest age cohorts (*p*≤.0012). For the 67C block, the CE did not differ significantly between age groups (*p*=.83). Finally, for the 47C block, the CE was significantly larger in young adults (*M*=1.64%) compared to the older age groups ($M_{60\text{-}69}$=0.57%, $M_{70\text{-}79}$=-0.46%, M_{80+}=0.11%; all *p*<.001). Moreover, the CE was significantly smaller in the 70-69 group compared to the 60-69 and 80+ groups (*p*≤.042). In general, the CE was larger in young adults compared to older adults, especially for the 47C block. Moreover, the CE in the 80+ group was larger than the other age groups for most blocks.

Addressing speed-accuracy trade-offs

Controlling for reaction time in the generalized linear mixed models

To account for possible speed-accuracy trade-offs, we included and thereby controlled for reaction time (or list completion time for the Plus-Minus task) in the generalized linear models with error rate as dependent measure for each of the tasks (e.g. in line with Davidson & Martin, 2013). To limit complexity and increase chances of model convergence, a variable containing mean RT per participant for each task was calculated and this variable was then added as a fixed main effect in the models, to control for the overall RT in a given task.

Age differences in inhibition.

Go/No-Go task. The GLMM for errors showed a main effect of Age Group (X^2(3)=62.61, *p*<.001). Post-hoc comparisons showed that the 60-69 group (*M*=4.98%) made less errors than the 80+ groups (*M*=7.20%; *Z*=-2.79, *p*=.028, *d*=-2.78). In addition, a main effect of RT was observed (X^2(1)=43.52, *p*<.001). One unit increase on the standardized RT scale corresponded to an increase of 0.50 in error rates, indicating that for longer RTs, more errors were made. Moreover, a main effect of

GoNoGo was found ($X^2(1)=494.55$, $p<.001$). More errors were made on No Go (M=8.98%) compared to Go trials (M=4.03%). In addition, a significant interaction between GoNoGo and Group was observed ($X^2(3)=604.04$, $p<.001$). For Go trials, significantly more omission errors were made with older age ($M_{young\ adults}$=2.36%, $M_{60\text{-}69}$=3.09%, $M_{70\text{-}79}$=4.44%, M_{80+}=7.96%, $p\leq.026$, d=-0.38-1.28), except for the young adults vs. the 60-69 group (p=.47). For No Go trials, young adults (M=21.70%) made significantly more commission errors than the older adult groups ($M_{60\text{-}69}$=7.94%, $M_{70\text{-}79}$=5.37%, M_{80+}=6.51%, all $p<.001$, d=1.17-1.59). In addition, the 70-79 group made less commission errors than the 60-69 group (p=.026, d=0.42). The other comparisons were not significant ($p>.52$).

Flanker task. The GLMM for errors showed a significant main effect of Congruency ($X^2(1)=6.47$, p=.011), with participants making more errors on incongruent (M=1.90%) than congruent trials (M=1.57%), i.e. the congruency effect. Post-hoc comparisons for the main effect of Age Group ($X^2(3)=56.58$, $p<.001$) showed that young adults (M=4.34%) made more errors on the Flanker task than the 60-69 (M=0.92%; Z=8.81, $p<.001$, d=1.59), 70-79 (M=1.45%; Z=6.59, $p<.001$, d=1.12) and 80+ groups (M=1.52%; Z=5.45, $p<.001$, d=1.08). Older adults between 60 and 69 made less errors than the 70-79 (Z=-2.59, p=.047, d=-0.46) and 80+ group (Z=-2.67, p=.038, d=-0.51). There were no significant differences in errors between older adults aged 70 to 79 and 80 years or older (p=.99). The significant interaction effect between Congruency and Age Group ($X^2(3)=14.56$, p=.0022) was further explored by comparing the congruency effect between Age Groups ($F(3,302)=193.7$, $p<.001$). More specifically, the CE was larger in young adults (M=1.68%) compared to all older adult cohorts (M_{60-69} =0.56%, M_{70-79} =-0.46%, M_{80+}=0.37%, $p\leq.001$, $d\geq1.65$). In addition, the CE of the 70-79 group was smaller compared to that of the other older age cohorts (all $p<.001$, $d\geq-2.09$). There were no significant differences in CE between the 60-69 and 80+ group (p=.18). No main effect of RT was found (p=.24).

Age differences in updating.

N-Back task. The GLMM for errors showed a main effect of Age Group ($X^2(3)=117.91$, $p<.001$). More errors were made on this task with increasing age. Young adults (M=6.34%) made less

errors than the 60-69 (M=12.59%; Z=-6.40, p<.001, d=-0.76), 70-79 (M=17.51%; Z=-9.21, p<.001, d=-1.14) and 80+ age groups (M=18.80%; Z=-9.16, p<.001, d=-1.23). Moreover, the 60-69 group showed less false alarms than the 70-79 (Z=-3.76, p=.0010, d=-0.39) and 80+ group (Z=-4.27, p<.001 , d=-0.47). There were no significant differences between the two oldest age groups (p=.84). In addition, a main effect of Target was observed ($X^2(3)$=758.98, p<.001). More errors were made on target trials (M=20.06%, i.e. missed targets) compared to non-target trials (M=8.02%, i.e. false alarms). Moreover, a main effect of RT was observed ($X^2(1)$=10.91, p<.001). One unit increase on the standardized RT scale corresponded to an increase of 0.15 in error rates, indicating that for longer RTs, more errors were made. Finally, a significant interaction between Hit and Group was observed ($X^2(3)$=21.31, p<.001). For both omission errors or missed targets and false alarms, more errors were made with older age (for missed targets: $M_{\text{young adults}}$=12.02%, $M_{60\text{-}69}$=19.27%, $M_{70\text{-}79}$=24.71%, M_{80+}=27.02%, p≤.030, d≥-0.32; for false alarms: $M_{\text{young adults}}$=3.24%, $M_{60\text{-}69}$=8.00%, $M_{70\text{-}79}$=12.08%, M_{80+}=12.65%, all p≤.001, d≥-0.46). No significant differences in both error types were observed between the two oldest age groups (p≥.73).

Age differences in shifting.

***Plus-Minus task*.** The GLMM for total number of errors showed a significant main effect of Switch ($X^2(1)$=12.97, p<.001). Participants made more errors on the switch block (M=0.42 errors or 1.40%) compared to the non-switch blocks (M=0.69 errors or 2.30%), indicating a switch cost. Moreover, a main effect of List completion time was observed ($X^2(1)$=28.57, p<.001). A positive slope of 0.38 was observed, indicating that participants made more errors with slower list completion times. All other effects were not significant (p≥.16).

Age differences in proactive and reactive control.

AX-CPT. The GLMM for error rates showed a significant main effect of Trial Type ($X^2(3)$=483.26, p<.001). Most of the errors were made on AY trials (M=5.61%), followed by BX (M=3.83%), BY (M=2.52%) and AX trials (M=1.91%, all p<.001, d=0.40-1.11). In addition, a main effect

of Group was found (X^2(3)=14.05, *p*=.0028). Young adults (*M*=6.08%) made significantly more errors on the task compared to all older adult groups (M_{60-69}=2.16%, M_{70-79}=2.82%, M_{80+}=2.80%, *p*≤.033, *d*≥.80). Moreover, a main effect of RT was observed (X^2(1)=26.70, *p*<.001). One unit increase on the standardized RT scale corresponded to an increase of 0.52 in error rates, indicating that for longer RTs, more errors were made. We also observed an interaction between Trial Type and Age Group (X^2(9)=54.04, *p*<.001). Importantly, for all Age Groups performance on AY trials was always worse than BX trials, but this was only significantly for young adults and the 70-79 group (*p*≤.0022), indicating a proactive control strategy. Moreover, for AY trials, young adults (*M*=13.76%) made significantly more errors than the older adult groups (M_{60-69}=3.35%, M_{70-79}=4.73%, M_{80+}=4.36%, all *p*<.001, *d*=1.25-1.53). Similarly, for BX trials, young adults (*M*=8.74%) made significantly more errors than the older adult groups (M_{60-69}=2.42%, M_{70-79}=2.88%, M_{80+}=3.45%, all *p*≤.012, *d*=0.99-1.35). No differences between the other age groups were observed (*p*≥.52).

***Flanker task with proportion congruency manipulation*.** The GLMM for the errors showed a significant main effect of Age Group (X^2(3)=48.82, *p*<.001), showing that young adults made significantly more errors (*M*=3.79%) compared to older adults of 60-69 (*M*=1.14%, *Z*=8.23, *p*<.001, *d*=1.23), 70-79 (*M*=1.53%, *Z*=6.28, *p*<.001, *d*=0.93) and 80 years or older (*M*=1.63%, *Z*=5.12, *p*<.001, *d*=0.87). There were no significant differences in errors between the older age groups (*p*≥.094). All other main effects were not significant (*p*≥.17). Regarding the interaction effect between Block and Age Group (X^2(6)=20.01, *p*=.0028), pairwise comparisons showed a significant difference in errors between blocks only for the older adults aged 60-69 and 80+. For the 60-69 group, significantly more errors were made on the 67C block (*M*=1.37%) compared to the 47C block (*M*=0.94%, *Z*=3.22, *p*=.0037, *d*=-0.39). For the 80+ group, significantly more errors were made on the 87C block (*M*=2.15%) compared to the 67C (*M*=1.51%, *Z*=3.97, *p*<.001, *d*=-0.53) and 47C block (*M*=1.43%, *Z*=2.58, *p*=.027, *d*=-0.32). Moreover, whereas for all blocks young adults always performed worse than older adults (all *p*<.001), older adults aged 80+ performed significantly worse than older adults aged 60-69 only for the 47C (M_{60-69} =0.94%, M_{80+}=1.56%, *Z*=2.85, *p*=.023, *d*=-0.52) and 87C (M_{60-69}

=1.16%, M_{80+}=2.15%, *Z*=3.11, *p*=.010, *d*=-0.63) blocks. Studying the interaction effect between Congruency and Age Group (X^2(3)=11.85, *p*=.0079), young adults (*M*=1.58%) had a significantly larger CE than older adults (M_{60-69} =0.47%, M_{70-79} =0.33%, M_{80+}=0.43%), indicating a worse inhibition (all *p*≤.001). The differences in CE between the older adults were not significant (*p*≥.91). For the interaction between Block and Congruency (X^2(2)=8.24, *p*=.016, i.e., a PCE), CEs were compared between blocks. With increasing proportion of incongruent trials, the CE became significantly smaller ($M_{87\%C}$ =1.44%, $M_{67\%C}$=0.77%, $M_{47\%C}$ =0.46%, *p*≤.001), i.e. the PCE. The significant three-way interaction between Block, Congruency and Age Group (X^2(6)=17.34, *p*=.0081) was further explored by comparing the PCE between Age Groups. The PCE was larger in the older adults aged 70-79 (*M*=1.79%) compared to young adults (*M*=1.24%, *p*=.0011, *d*=-0.52), older adults aged 60-69 (*M*=-0.31%, *p*<.001, *d*=-2.26) and 80+ (*M*=1.29%, *p*=.0069, *d*=0.43). Moreover, the PCE was smaller in the 60-69 group compared to young adults (*p*<.001, *d*=2.87) and the 80+ group (*p*<.001, *d*=-2.43). There was no significant difference between the young adults and 80+ group (*p*=.98).

In general, when controlling for overall RT, the observed age effects remained highly similar. Moreover, no evidence was found for a speed-accuracy trade-off across tasks. Across age groups, a positive slope was observed for all tasks (except the Flanker task), showing that the slower RTs were accompanied by higher error rates, contradicting a typical speed-accuracy trade-off pattern.

Comparing the Inverse Efficiency Scores

In addition to controlling for RT in the error rate analyses as reported in the previous section, we also calculated an Inverse Efficiency Score (IES; Townsend and Ashby, 1983) for each task, combining speed and accuracy in a single measure. This index can be interpreted as "the time spent for correct responses" (Statsenko et al., 2020, p. 3), as the mean RT on a task is corrected by accuracy on the task.

More specifically, for the Go/No-Go task, the IES was calculated by dividing the mean RT on correct Go trials by (100 – the mean ratio of commission errors), multiplied by 100. For the N-Back

task, the mean RT on correct target trials was divided by (100 – the mean ratio of missed target errors), multiplied by 100. For all other tasks, mean RT on correct trials across conditions (or mean list completion time across lists for the Plus-Minus task) was divided by (100 – mean ratio of errors across conditions) multiplied by 100. These scores were then compared between groups using one-way ANOVAs with Tukey correction for multiple comparisons.

Age differences in inhibition.

Go/No-Go task. The IES differed significantly between groups ($F(3, 302)=37.70$, $p<.001$). Young adults ($M_{\text{Young adults}}=336$ms) showed a smaller IES compared to older adults ($M_{60\text{-}69}=400$ms, $M_{70\text{-}79}=408$ms, $M_{80+}=433$ms; all $p<.001$). Moreover, the IES was significantly larger in the 80+ group compared to the 60-69 ($p=.0030$) and 70-79 groups ($p=.035$). No significant differences were observed between the 60-69 and 70-79 groups ($p=.84$).

Flanker task. The IES differed significantly between groups ($F(3, 302)=21.67$, $p<.001$). Young adults ($M_{\text{Young adults}}=595$ms) showed a smaller IES compared to older adults ($M_{60\text{-}69}=725$ms, $M_{70\text{-}79}=742$ms, $M_{80+}=845$ms; all $p<.001$). Moreover, the IES was significantly larger in the 80+ group compared to the 60-69 ($p<.001$) and 70-79 groups ($p=.0050$). No significant differences were observed between the 60-69 and 70-79 groups ($p=.94$).

Age differences in updating.

N-Back task. The IES differed significantly between groups ($F(3, 292)=28.96$, $p<.001$). The IES was smaller in young adults ($M_{\text{Young adults}}=715$ms) compared to older adults ($M_{60\text{-}69}=989$ms, $M_{70\text{-}79}=1201$ms, $M_{80+}=1310$ms; all $p<.001$). Moreover, the IES was significantly smaller in the 60-69 group compared to the 70-79 ($p=.0080$) and 80+ groups ($p<.001$). No significant differences were observed between the 70-79 and 80+ groups ($p=.39$).

Age differences in shifting.

Plus-Minus task. The IES differed significantly between groups ($F(3, 297)=36.02$, $p<.001$). Young adults ($M_{\text{Young adults}}=103$s) showed a smaller IES compared to older adults ($M_{60\text{-}69}=138$s, $M_{70\text{-}}$

$_{79}$=190s, M_{80+}=196s; $p≤.0036$). Moreover, the IES was significantly smaller in the 60-69 group compared to the 70-79 and 80+ groups (all $p<.001$). No significant differences were observed between the 70-79 and 80+ groups ($p=.95$).

Age differences in proactive and reactive control.

AX-CPT. The IES differed significantly between groups ($F(3, 288)=21.65$, $p<.001$). Young adults ($M_{\text{Young adults}}$=487) showed a smaller IES compared to older adults ($M_{60\text{-}69}$=644, $M_{70\text{-}79}$=679, M_{80+}=759; all $p<.001$). Moreover, the IES was significantly smaller in the 60-69 group compared to the 80+ groups ($p=.035$). No significant differences were observed between other groups ($p≥.096$).

Flanker task. The IES differed significantly between groups ($F(3, 302)=32.73$, $p<.001$). Young adults ($M_{\text{Young adults}}$=577) showed a smaller IES compared to older adults ($M_{60\text{-}69}$=717, $M_{70\text{-}79}$=728, M_{80+}=853; all $p<.001$). Moreover, the IES was significantly larger in the 80+ group compared to the 60-69 and 70-79 groups (all $p<.001$). No significant differences were observed between the 60-69 and 70-79 groups ($p=.97$).

To summarize, after controlling for errors on each task and in line with the original RT analyses, age-related patterns of slowing remained present across the different cognitive control components.

Variability as an index of cognitive performance

In the main manuscript we looked at the effect of age on RTs and errors in different cognitive control tasks, as a measure of cognitive control performance. By using (generalised) linear mixed models, and adding random intercepts for participants and slopes for conditions, we took into account intra-individual variability. However, variability in itself could also be an indicator of age-related changes in cognitive performance (MacDonald et al., 2006). Hence, to explore and illustrate this variability, we conducted one-way ANOVAs and mixed ANOVAs comparing intra-individual variability between groups and conditions. Variability was indexed here as the standard deviation of

RTs for each task. In addition, for the Go/No-Go and N-Back tasks we also studied and compared standard deviations for commission errors and missed targets, as these are the main indices for inhibition and updating respectively.

More specifically, for the Go/No-Go and N-Back tasks, one-way ANOVAs were conducted with the *SD* of RTs on correct trials and the *SD* of commission errors or missed targets respectively as outcome variables and Group as factor. For all other tasks, the *SD* of RTs of correct trials was analysed with repeated measures ANOVAs with Group as factor and Congruency (for the Flanker task for inhibition), Trial type (for the AX-CPT) or Congruency and Block (for the Flanker with three blocks for proactive and reactive control). For the Plus-Minus task we only had one speed-related index per list (i.e. list completion time), so variability within lists could not be studied. Hence, one-way ANOVAs for variability in time to complete a list was compared across the three lists between groups. These analyses were conducted using SPSS v.29.0.1.0 (the script and dataset are openly shared on the OSF page, https://osf.io/vbh58/). Significance levels were manually adjusted for multiple comparisons using Bonferroni correction (α/number of comparisons).

Age differences in inhibition

Go/No-Go task. Variability, as indexed by the *SD* of the Go/No-Go RTs, differed significantly between groups ($F(3, 302)=16.25$, $p<.001$). Young adults (M=45ms) showed a smaller variability compared to older adults aged 70 or older (M_{70-79}=49ms, M_{80+}=57ms; $p\leq.008$, d=-.43 – -1.09). Moreover, variability was significantly larger in the 80+ group compared to the 60-69 (M_{60-69}=47.58) and 70-79 groups (all $p<.001$, d=-.63 – -.84). No significant differences were observed between the other groups ($p\geq.092$).

Variability, as indexed by the *SD* of the commission errors, differed significantly between groups ($F(3, 302)=5.48$, $p<.001$). Young adults (M=31.51%) showed a larger variability compared to older adults aged 60-69 (M_{60-69}=26.08%, p=.007, d=.44) and 70-79 (M_{70-79}=23.51%; $p<.001$, d=.64). No significant differences were observed between the other groups (M_{80+}=28.49%; all $p\geq.025$).

Flanker task. A significant main effect of Congruency was observed ($F(1, 302)=7.33$, $p=.007$). The variability, as indexed by the *SD* of the Flanker RTs in the 47C block, was larger in congruent trials (M=148ms) compared to incongruent trials (M=138ms). Moreover, a significant main effect of Group was found ($F(3, 302)=4.92$, $p=.002$). Young adults (M=127ms) showed a smaller variability compared to older adults of the 80+ group (M_{80+}=181ms; $p=.003$, $d=-.51$). Moreover, variability was significantly smaller in the 60-69 group ($M_{60\text{-}69}$=133ms) compared to the 80+ group ($p=.006$, $d=-.48$). No significant differences were observed between the other groups (all $p \geq .034$). No interaction effect between Congruency and Group was found ($p=.35$).

Age differences in updating

N-Back task. Variability, as indexed by the *SD* of the N-Back RTs, differed significantly between groups ($F(3, 292)=3.27$, $p=.022$). Young adults ($M_{\text{young adults}}$=194ms) showed a smaller variability compared to older adults aged 80 or older (M_{80+}=220ms; $p=.008$, $d=-.46$). No significant differences were observed between the other groups (all $p \geq .010$).

Variability, as indexed by the *SD* of the missed target errors, differed significantly between groups ($F(3, 292)=25.97$, $p<.001$). Young adults ($M_{\text{young adults}}$=29.37%) showed a smaller variability compared to older adults ($M_{60\text{-}69}$=37.43%, $M_{70\text{-}79}$=41.45%, M_{80+}=42.45%; all $p<.001$, $d=-.43$ – -1.22). Moreover, the 60-69 group showed a smaller variability than the 70-79 group ($p=.008$) and 80+ group ($p=.004$). No significant differences were observed between the two oldest groups ($p=.52$).

Age differences in shifting

Plus-Minus task. Variability, as indexed by the *SD* of the Plus-Minus list completion times across lists, differed significantly between groups ($F(3, 297)=14.36$, $p<.001$). Variability increased with age. Young adults ($M_{\text{young adults}}$=12s) showed a smaller variability compared to older adults ($M_{60\text{-}69}$=21s, $M_{70\text{-}79}$=35s, M_{80+}=31s; $p \leq .001$, $d=-.53$ – -1.06). In addition, the 60-69 group showed smaller variability than the 70-79 group ($p=.001$, $d=-.53$). No significant differences were observed between the other older adult groups after correcting for multiple comparisons (all $p \geq .025$).

Age differences in proactive and reactive control

AX-CPT. A significant main effect of Trial type was observed ($F(3, 286)=51.43$, $p<.001$). The variability, as indexed by the *SD* of the AX-CPT RTs, was largest for BX trials (M=166ms), followed by BY (M=149ms), AX (M=134ms) and AY trials (M=125ms). All comparisons were significant (all $p<.001$, d=.24-.72). Moreover, a significant main effect of Group was found ($F(3, 288)=3.21$, $p=.024$). Older adults of the 80+ group (M_{80+}=175ms) showed a larger variability compared to young adults ($M_{young\ adults}$=146ms; $p=.006$, d=-.48) and the 60-69 group ($M_{60\text{-}69}$=144ms; $p=.003$, d=-.53). No significant differences were observed between the other groups ($M_{70\text{-}79}$=157ms; all $p\geq.072$). No interaction effect between Trial type and Group was found ($p=.11$).

Flanker task. A significant main effect of Block was observed ($F(2, 288)=3.97$, $p=.020$). The variability, as indexed by the *SD* of the Flanker RTs, was larger in the 87C block (M=149ms), compared to the 67C block (M=138ms, $p<.001$, d=-.21). The other pairwise comparisons for Block were not significant after correcting for multiple comparisons (all $p\geq.037$). Moreover, a significant main effect of Congruency was found ($F(1, 289)=22.87$, $p<.001$). Variability was larger in congruent (M=149ms) compared to incongruent trials (M=141ms). In addition, a main effect of Group was observed ($F(3, 289)=6.19$, $p<.001$). The 80+ group (M_{80+}=187ms) showed a larger variability compared to young adults ($M_{young\ adults}$=127ms, $p=.001$, d=-.59) and the 60-69 ($M_{60\text{-}69}$=140ms, $p=.006$, d=-.47). No significant differences were observed between the other groups ($M_{70\text{-}79}$=143ms; all $p\geq.015$). Finally, the interaction between Block and Group was significant ($F(6,576)=2.18$, $p=.043$). Only for the 80+ group, a significant difference was observed between variability in the 87C (M=197ms) and 47C block (M=169ms; $p=.007$, d=-.35). No other differences were detected ($p\geq.019$). When looking at group differences per block, variability was always larger in the 80+ group compared to young adults (all $p\leq.006$, d=-.48 – -.66). In addition, only for the 67C and 87C block, variability was larger in the 80+ group compared to the 60-69 and 70-79 group (all $p\leq.008$, d=-.46 – -.54). No other interaction effects were found (all $p\geq.22$).

To summarize, in general, intra-individual variability for all measures except the commission errors was larger with older age. With older age, performance thus seemed to fluctuate more across trials.

REFERENCES

Davidson, D. J., & Martin, A. E. (2013). Modeling accuracy as a function of response time with the generalized linear mixed effects model. *Acta psychologica*, *144*(1), 83-96. https://doi.org/10.1016/j.actpsy.2013.04.016

Townsend, J. T., & Ashby, F. G. (1983). *Stochastic modeling of elementary psychological processes*. CUP Archive.

Statsenko, Y., Habuza, T., Gorkom, K. N.-V., Zaki, N., & Almansoori, T. M. (2020). Applying the Inverse Efficiency Score to Visual–Motor Task for Studying Speed-Accuracy Performance While Aging. *Frontiers in Aging Neuroscience*, *12*, 574401. https://doi.org/10.3389/fnagi.2020.574401

MacDonald, S. W. S., Nyberg, L., & Bäckman, L. (2006). Intra-individual variability in behavior: Links to brain structure, neurotransmission and neuronal activity. *Trends in Neurosciences*, *29*(8), 474–480. https://doi.org/10.1016/j.tins.2006.06.011
